# Crimean-congo hemorrhagic fever in Iraq, 2021–2024: epidemiological and clinical data analysis with proposed severity indicators for resource-constrained settings

**DOI:** 10.1186/s12879-026-12759-z

**Published:** 2026-02-02

**Authors:** Chiori Kodama, Walaa Ismail, Riyadh Abdulameer Alhilfi, Ihab Aakef, Hameeda Mohammed Hasan, Ghazwan A. Baghdadi, Raghad Ibrahim Khaleel, Anais Legand, Olivia Keiser, Isabella Eckerle, Pierre B.H. Formenty, Adnan Khamasi, Sinan Mahdi

**Affiliations:** 1 Former World Health Organization, Eastern Mediterranean Regional Office, Cairo, Egypt; 2 Institute of Global Health, University of Geneva, Geneva, Switzerland; 3 Japan Institute for Health Security, Tokyo, Japan; 4 Directorate of Public Health, Ministry of Health, Baghdad, Iraq; 5 Communicable Disease Control Center, Ministry of Health, Baghdad, Iraq; 6 Central Public Health Laboratory, Ministry of Health, Baghdad, Iraq; 7 World Health Organization, Headquarters, Geneva, Switzerland; 8 Faculty of Medicine, Geneva Centre for Emerging Viral Diseases, University Hospitals of Geneva, University of Geneva, Geneva, Switzerland; 9 Former World Health Organization, Headquarters, Geneva, Switzerland; 10 Former World Health Organization, Country Office for Iraq, Baghdad, Iraq

**Keywords:** Crimean-Congo hemorrhagic fever, Iraq, Viral hemorrhagic fever, Severity scoring system, High-risk subgroup, Clinical decision-making, Epistaxis, Jaundice, Platelet, Ct-value, Resource-constrained settings

## Abstract

**Background:**

Crimean–Congo hemorrhagic fever (CCHF) is a high-fatality zoonotic infection. Iraq has experienced a substantial increase in reported CCHF cases since 2021, yet predictors of mortality and the feasibility of existing severity scores in routine care have not been systematically assessed. We aimed to identify demographic, clinical and laboratory predictors of death among confirmed CCHF patients in Iraq and to develop a simplified severity score suitable for bedside use in resource-constrained settings.

**Methods:**

We analyzed 273 laboratory-confirmed CCHF cases with known outcomes reported to the Iraqi national surveillance system from 1 January 2021 to 31 December 2024 (273/1,193 confirmed cases with complete standardized forms). Demographic, exposure, clinical, and laboratory variables (including platelet count and cycle threshold [Ct] values) were extracted from harmonized Ministry of Health and Central Public Health Laboratory databases. Univariable and multivariable logistic regression were used to identify mortality predictors. Receiver Operating Characteristic (ROC) curve analysis compared the discriminative performance (area under the curve, AUC) of newly proposed scores with existing CCHF Severity Scoring Index (SSI) and Swanepoel’s Grading Score (SGS).

**Results:**

The overall case fatality rate was 12.1% (33/273). Age, sex, occupation, place of residence, and exposure history were not significantly associated with mortality. In univariable analysis, low platelet count (< 20 × 10³/µL), high viral load (Ct ≤ 25), epistaxis, gastrointestinal bleeding, any bleeding, and jaundice were associated with increased odds of death; after false discovery rate correction, epistaxis, gastrointestinal bleeding, any bleeding, and jaundice remained significant. In multivariable analysis, only epistaxis retained an independent association with mortality (adjusted OR 4.70; 95% CI 1.08–20.4). A laboratory-enhanced three-variable score (epistaxis, low platelet count, Ct ≤ 25) achieved an AUC of 0.70, similar to SSI (0.69). A two-variable score (epistaxis + low platelet count) had an AUC of 0.69, and a purely clinical score (epistaxis, any bleeding, jaundice) an AUC of 0.62, close to SGS (0.64).

**Conclusion:**

Epistaxis and jaundice emerged as pragmatic clinical red-flag indicators of poor outcome, with low platelet count and high viral load supporting risk stratification where available. Very simple, clinically based scores performed comparably to existing complex tools and may facilitate earlier triage and intensified supportive care for high-risk CCHF patients in resource-limited settings.

**Supplementary Information:**

The online version contains supplementary material available at 10.1186/s12879-026-12759-z.

## Background

Crimean-Congo hemorrhagic fever (CCHF) is the most geographically widespread tick-borne disease, identified in more than 30 countries across Africa, Asia, the Middle East, and parts of Europe located south of the 50th parallel north [1, 2]. Crimean-Congo hemorrhagic fever virus (CCHFV) causes severe viral hemorrhagic fever outbreaks with a case fatality rate (CFR) ranging from 5% to 40% [1–5]. Globally, an estimated three billion people are at risk, and annual incidence estimates range from 10,000 to 15,000 cases, with a slow yet steady increase. Most infections result from tick bites, followed by exposure through bodily fluids or tissues from infected animals, and, less commonly, human-to-human transmission within household or healthcare settings. CCHF presents a significant global health threat due to its epidemic potential, high fatality rate, and absence of specific treatments or vaccines [1–6].

In recent years, Iraq has been among the countries most severely affected by CCHF [6, Supplementary Material 1]. We recently conducted epidemiological research examining potential factors influencing the 2022–2023 CCHF outbreak in the country [6]. While the national CCHF case-management protocol was expanded in 2021, its clinical implementation and outcome predictors have not been systematically evaluated. Strengthening One Health collaboration could improve the understanding of disease prevalence and the association between human and animal cases, while enhanced assessment and diagnostic capacity would improve clinical management and potentially reduce mortality. Although CCHF severity scoring systems such as the Crimean-Congo Hemorrhagic Fever Severity Scoring Index (CCHF-SSI) [7], which uses clinical and laboratory parameters to stratify disease severity and prognosis, and Swanepoel’s Grading Score (SGS) [8], which categorizes severity based on clinical and laboratory findings, are available, their implementation remains challenging in resource-limited settings due to insufficient laboratory infrastructure, lack of equipment, and cost constraints. While some studies have validated the efficacy of these severity scores [9, 10], the unavailability of key indicators in field settings often hampers their application. Consequently, this leads to delayed clinical screening and management, resulting in increased mortality. Therefore, it is crucial to identify practical and simplified CCHF severity indicators that can enable timely clinical decision-making, effective patient management, and improved prediction of disease outcomes in resource-limited environments.

## Objectives

This study (i) identifies demographic, clinical and laboratory predictors of mortality among confirmed CCHF patients in Iraq (2021–2024); and (ii) develops and compares a simplified severity score suitable for bedside use.

## Methods

### Data source and collection

Study data were extracted from the national surveillance system and standardized case reporting forms (Excel, Microsoft) [Supplementary material 2], jointly developed and implemented by the Ministry of Health Iraq and the World Health Organization. Standardized case reporting forms were developed using Epi Info version 7.2.5 (April 2024) to systematically collect clinical data, including patient demographics, exposure history, clinical outcomes, laboratory-confirmed diagnoses, blood test results, administered treatments, and other relevant clinical parameters. The original data collection form, along with the complete list of indicators, is provided in the supplementary materials (Supplementary Material 2). These forms were systematically used to collect data from all study sites (designated CCHF hospitals). Data collection using standardized forms commenced in July 2024, immediately following training sessions provided to data collectors assigned at each study site. Consequently, data collected prior to July 2024 were gathered retrospectively, while data collected thereafter were gathered prospectively. Using this approach, complete datasets were obtained for 273 (23%) out of a total of 1,193 confirmed CCHF cases reported in Iraq between 2021 and 2024. Suspected cases from this period were excluded from the study due to observed inconsistencies in applying the suspected case definition. Cases with missing outcome data were excluded; incomplete laboratory variables (e.g., Ct value) were retained as NA entries.

### Study population

The study population included individuals confirmed with CCHF and reported to the Ministry of Health Iraq/Communicable Disease Control Center/Central Public Health Laboratory from 1 January 2021 to 31 December 2024. All suspected and confirmed CCHF cases were admitted to designated general hospitals - at least one designated CCHF hospital was preliminarily assigned in each governorate - and managed within infectious disease wards and intensive care units at these respective hospitals.

### Laboratory diagnostics and recording

Laboratory confirmation of CCHFV infection was performed by the Iraq Central Public Health Laboratory (CPHL) using real-time reverse transcription polymerase chain reaction (RT-PCR; Altona Diagnostics RealStar^®^ CCHFV RT-PCR Kit) and/or IgM enzyme-linked immunosorbent assay (ELISA; VectoCrimean-CHF-IgM, Vector-Best, Russia), according to national guidelines. Cycle threshold (Ct) values were recorded during RT-PCR testing for 138 confirmed CCHF cases, representing 11% of the total confirmed cases during 2021–2024, and 51% of the 273 cases included in this study. Ct values are inversely proportional to viral load, with low Ct values indicating high viral load [11–14]. Previous studies [13, 14] suggest the classification of Ct values for CCHF as follows: high viral load (Ct ≤ 25), moderate viral load (25 < Ct ≤ 35), and low viral load (Ct > 35).

### Data management

A total of 318 variables were collected, covering patient profiles - including comorbidities and medications, governorate of residence, potential risk factors, clinical manifestations, vital signs, patient outcomes, laboratory results including Ct-values (for patients with positive RT-PCR), and blood test results (e.g., complete blood count [CBC], platelet count, coagulation profiles, liver enzymes, electrolytes, and renal function tests) at the time of hospital admission and during follow-up examinations where applicable. To validate demographic information and accurately document disease outcomes, data from the CPHL database were manually matched and merged with data from the Ministry of Health’s epidemiological and clinical databases. Patient names and sample identifiers recorded in both databases served as primary identifiers for merging, with additional variables used to verify data accuracy and consistency.

### Variables and statistical analysis

Although a large number of variables were collected in the raw dataset, we selected those most relevant for accurate mortality risk based on data availability, clinical importance, and previous literature [15–17]. These included demographic characteristics; clinical symptoms (fever, hypotension/shock, petechiae, epistaxis, gastrointestinal bleeding, bleeding from injection sites, any bleeding signs, jaundice, and hepatomegaly); and laboratory results (platelet count and Ct values). Although altered mental status was recorded for all patients, its assessment lacked standardized criteria and relied on subjective clinical judgment; therefore, it was excluded from regression analyses focused on reproducible bedside indicators.

All statistical analyses were performed using R software (version 4.4.2; R Foundation for Statistical Computing, Vienna, Austria). Statistical significance was defined as *p* < 0.05. Descriptive analyses were first conducted for the 273 laboratory-confirmed CCHF cases to select the variables to proceed to further study. Univariable logistic regression was used to assess associations between individual variables and mortality. Variables with significant associations (*p* < 0.05) or clear clinical relevance were entered into a multivariable logistic regression model. Continuous variables such as platelet count and Ct values were evaluated both as continuous measures and categorized using clinically meaningful cut-offs derived from prior studies [15]. Adjusted odds ratios (ORs) with 95% confidence intervals (CIs) were calculated and visualized using forest plots.

A geospatial analysis was additionally performed to visualize median intervals from symptom onset to hospital admission by governorate. Shapefiles defining Iraq’s administrative boundaries were obtained from publicly available sources [18].

To identify practical mortality-prediction indicators suitable for resource-constrained settings, we conducted additional logistic regression analyses focusing exclusively on clinically accessible severity markers. Based on significant multivariable predictors, a simplified severity scoring system was developed. Its discriminative performance was evaluated using Receiver Operating Characteristic (ROC) curve analysis, performed in Python, and compared against existing SSI and SGS scores.

### Ethical considerations

Human case report data were anonymized after merging additional laboratory information into the dataset, under the supervision of the Ministry of Health of Iraq, and with appropriate ethical approval obtained.

## Results

Data completeness for key laboratory variables varied substantially, ranging from 250 cases (94%) for platelet count to 138 cases (50.5%) for Ct value and 26 cases (9.5%) for creatinine. Subsequent regression analyses for each variable, along with the development of a practical and simplified set of CCHF severity indicators and the rationale for inclusion or exclusion of variables, are described in the Methods section (“Variables and Statistical Analysis”) and in the subsequent Results and Discussion sections.

### Risk factors associated with mortality among confirmed CCHF cases

After an initial analysis of the 318 collected variables, data presented in Supplementary Material 3 indicate that demographic characteristics, including age and gender, were not significantly associated with mortality. The median age (IQR) did not significantly differ between survivors and non-survivors. Male patients had lower odds of mortality compared to females; however, this association did not reach statistical significance. Regarding place of residence, there were no statistically significant differences in mortality observed when comparing patients from semi-urban areas or urban areas to those residing in rural areas. Occupational categories, including butchers, animal owners, and homemakers, also showed no statistically significant association with increased mortality risk. Similarly, exposure history variables, such as the presence of animals at home, history of tick bites, presence of ticks on animals or in the home environment, history of animal slaughtering, and handling raw meat were not significantly associated with mortality. The presence of comorbidities was associated with lower odds of mortality, although this association did not achieve statistical significance.

Based on the collected data, a table and a map (Supplementary Material 4 − 1, 2) were generated to analyze the median time from symptom onset to hospital admission by governorate. Notable variations were observed, with the median number of days from symptom onset to admission ranging from 1 day to 14 days across different governorates. Further, the time-to-admission analysis suggests that longer delays were associated with higher case-fatality rates (CFRs) (Supplementary Material 4 − 3). Specifically, hospital admissions occurring ≥ 10 days after symptom onset had the highest observed CFR at 22.2% (male CFR: 16.7%; female CFR: 33.3%). Although this trend indicates that delays exceeding 10 days may substantially increase mortality risk - particularly among females - the differences in CFR across admission-delay categories were not statistically significant. While onset-to-admission delay appears to influence both symptom presentation and mortality risk, this variable was not included in further analyses, as the primary aim of this study was to identify clinical and basic laboratory predictors of mortality among confirmed CCHF patients in Iraq and to develop and compare a simplified severity score suitable for bedside use.

Among the laboratory indicators, platelet count and Ct value showed significant associations with mortality (Table 1). The lowest platelet category (< 20 × 10³/µL) demonstrated the highest CFR (20.5%), compared with 11.9% and 6.1% in the 20–49 × 10³/µL and ≥ 50 × 10³/µL groups, respectively (*p* = 0.022). Similarly, patients with higher viral loads (Ct ≤ 25) had a CFR of 19.3%, compared with 7.9% in the Ct 26–35 category. Although Ct > 35 also showed elevated mortality (20%), this estimate was based on only five patients. Ct-value remained statistically associated with mortality (*p* = 0.047), but interpretation is limited by substantial missing data (51.6%).

**Table 1:** Laboratory and clinical data analysis among 273 laboratory-confirmed CCHF cases in Iraq^a^

Laboratory data at the time of Hospital Admission		All *N* = 273	Survived *N* = 240		Death *N* = 33		CFR^b^		*p*-value^c, d^ overall
**Platelet count (×10³/µL)**		N*=273 (100%)	240		33		**12%**		**0.022**
Lower Platelets < 20 (x10³/µL)		73 (26.7%)	58 (24.2%)		15 (45.5%)		**20.5%**		
Medium Platelet 20–49 (x10³/µL)		101 (37.0%)	89 (37.1%)		12 (36.4%)		**11.9%**		
Higher Platelet ≥ 50 (x10³/µL)		82 (30.0%)	77 (32.1%)		5 (15.2%)		**6.1%**		
N/A*		17 (6.2%)	16 (6.7%)		1 (3.0%)		**5.9%**		
**Ct value**		N*=273 (100%)	240		33		**12%**		**0.047**
High viral load (Ct ≤ 25)		57 (20.9%)	46 (19.2%)		11 (33.3%)		**19.3%**		
Moderate viral load (Ct 26–35)		76 (27.8%)	70 (29.2%)		6 (18.2%)		**7.9%**		
Low viral load (Ct > 35).		5 (1.8%)	4 (1.7%)		1 (3.0%)		**20.0%**		
N/A*		135 (49.5%)	120 (50%)		15 (45.5%)		**11.1%**		

**Clinical Manifestation at the time of Hospital Admission**	**All** *N* = 273			**Survived** *N* = 240		**Death** *N* = 33		**p-value** ^**c, d**^ **overall**	
Fever (axillary temperature ≥ 38.0 °C)	N*=273 (100%)			240		33		0.633	
No (< 38.0 °C)	80 (29.3%)			73 (30.4%)		7 (21.2%)			
Yes (≥ 38.0 °C)	188 (68.9%)			163 (67.9%)		25 (75.8%)			
N/A*	11 (4.0%)			10 (4.2%)		1 (3.0%)			
Hypotension (SBP^e^ < 90 mm Hg)	N*=273 (100%)			240		33		0.065	
No (SBP ≥ 90㎜Hg)	257 (94.1%)			229 (95.4%)		28 (84.8%)			
Yes (SBP<90㎜Hg)	9 (3.3%)			6 (2.5%)		3 (9.1%)			
N/A*	13 (4.8%)			11 (4.6%)		2 (6.1%)			
Petechiae	N*=273 (100%)			240		33		0.065	
No	214 (78.3%)			193 (80.4%)		21 (63.6%)			
Yes	55 (20.1%)			44 (18.3%)		11 (33.3%)			
N/A*	4 (1.5%)			3 (1.3%)		1 (3.0%)			
Retro Orbital Pain	N*=273 (100%)			240		33		0.662	
No	260 (95.2%)			229 (95.4%)		31 (93.9%)			
Yes	13 (4.8%)			11 (4.6%)		2 (6.1%)			
**Epistaxis**	N*=269 (99%)			240		33		**< 0.001**	
No	232 (86.2%)			212 (88.3%)		20 (60.6%)			
Yes	37 (13.8%)			25 (10.4%)		12 (36.4%)			
N/A*	4 (1.5%)			3 (1.3%)		1 (3.0%)			
Bleeding from site of injection	N*=273 (100%)			240		33		0.212	
No	197 (72.2%)			177 (73.8%)		20 (60.6%)			
Yes	72 (26.4%)			60 (25.0%)		12 (36.4%)			
N/A*	4 (1.5%)			3 (1.3%)		1 (3.0%)			
Hematuria	N*=273 (100%)			240		33		0.399	
No	265 (97.1%)			234 (97.5%)		31 (93.9%)			
Yes	4 (1.5%)			3 (1.3%)		1 (3.0%)			
N/A*	4 (1.5%)			3 (1.3%)		1 (3.0%)			
**Gastrointestinal Bleeding**	N*=273 (100%)			240		33		**0.003**	
No	223 (81.7%)			203 (84.6%)		20 (60.6%)			
Yes	46 (16.8%)			34 (14.2%)		12 (36.4%)			
N/A*	4 (1.5%)			3 (1.3%)		1 (3.0%)			
Any bleeding signs*	N*=273 (100%)			240		33		**0.003**	
No	126 (46.2%)			119 (49.6%)		7 (21.2%)			
Yes	147 (53.8%)			121 (50.4%)		26 (78.8%)			
**Jaundice**	N*=273 (100%)			240		33		**0.014**	
No	268 (98.2%)			238 (99.2%)		30 (90.9%)			
Yes	5 (1.8%)			2 (0.8%)		3 (9.1%)			
Hepatomegaly	N*=273 (100%)			240		33		0.231	
No	259 (94.9%)			226 (94.2%)		33 (100%)			
Yes	14 (5.1%)			14 (5.8%)		0 (0%)			

a: Data source: Ministry of Health Iraq/Communicable Disease Control Center. *N* = 273 CCHF cases with known outcome, reported from 2021 to 2024

b: CFR=Case Fatality Rate

c: Comparisons were conducted using chi-square tests or Fisher’s exact tests for categorical variables and Student’s t-tests for continuous variables

d: A two-sided p-value of < 0.05 was considered statistically significant

e: SBP=Systolic Blood Pressure

N*=Actual number of collected data out of total 273 confirmed CCHF cases

N/A*=Data not available

Any bleeding signs*= ecchymosis, petechiae, bleeding from injection sites, epistaxis, bleeding gums, gastrointestinal bleeding, hematuria, vaginal bleeding, or other forms of hemorrhage

### Univariate analysis

Based on the selected laboratory and clinical indicators, univariate logistic regression analysis was performed (Table 2). Patients with low platelet counts (< 20 × 10³/µL) had nearly a four-fold higher odds of death compared with those with high platelet counts ≥ 50 × 10³/µL (OR 3.98, 95% CI 1.36–11.58; *p* = 0.012). Platelet counts of 20–49 × 10³/µL showed a non-significant trend toward increased mortality (OR 2.09, 95% CI 0.71–6.16; *p* = 0.180).

Ct value demonstrated a similar pattern. Patients with high viral load (Ct ≤ 25) had almost a three-fold higher odds of mortality compared to those with Ct 26–35 (OR 2.78, 95% CI 0.97–7.94; *p* = 0.056), although this association did not remain statistically significant after correction for multiple testing. The Ct > 35 category showed elevated mortality but with unstable estimates due to the very small sample size (*n* = 5). Platelet count remained statistically significant after False Discovery Rate (FDR) correction (q = 0.024), whereas the association for Ct value was borderline and limited by substantial missingness (q = 0.075).

Among clinical variables, epistaxis was associated with more than a five-fold increase in mortality (OR 5.09; 95% CI 2.23–11.6; *p* < 0.001), and gastrointestinal bleeding showed a greater than three-fold increase (OR 3.58; 95% CI 1.61–7.99; *p* = 0.002). When all bleeding manifestations were combined into a composite variable, any bleeding remained significantly associated with mortality (OR 3.47; 95% CI 1.39–8.70; *p* = 0.008). Among non-hemorrhagic features, jaundice showed the strongest association with death (OR 11.9; 95% CI 1.91–74.1; *p* = 0.008). After FDR correction, four variables - epistaxis, gastrointestinal bleeding, any bleeding, and jaundice - remained statistically significant (q < 0.05) (Table 2).

**Table 2:** Univariable logistic regression analysis of predictors associated with increased mortality among confirmed CCHF Cases^a^

Laboratory Variables at the time of Hospital Admission	OR^b^	95% CI^b^	*p*-value^c^	q-value^d, e^
**Low platelet count (< 20 × 10³/µL)**	**3.98**	**[1.36, 11.58]**	**0.012**	**0.024**
Medium platelet count (20–49 × 10³/µL)	2.09	[0.71, 6.16]	0.180	0.180
Higher platelet count (≥ 50 × 10³/µL)	1.00 (ref)	-	-	-
**High viral load (Ct ≤ 25)**	**2.78**	**[0.97**,** 7.94]**	**0.056**	**0.075**
Moderate viral load (Ct 26–35)	1.00 (ref)	-	-	-
Low viral load (Ct > 35).	2.92	[0.28, 30.70]	0.355	0.355

**Clinical Variables at the time of Hospital Admission**	**OR** ^**b**^	**95% CI** ^**b**^	**p-value** ^**c**^	**q-value** ^**d, e**^
Epistaxis (yes vs. no)	5.09	[2.23, 11.6]	0.0001	0.0009
Gastrointestinal bleeding (yes vs. no)	3.58	[1.61, 7.99]	0.002	0.009
Any bleeding signs (yes vs. no)	3.47	[1.39, 8.70]	0.008	0.018
Jaundice (yes vs. no)	11.90	[1.91, 74.11]	0.008	0.018

a: Data source: Ministry of Health Iraq/Communicable Disease Control Center. *N* = 273 CCHF cases with known outcome, reported from 2021 to 2024

b: OR = Odds Ratio; CI = Confidence Interval

c: Comparisons were conducted using chi-square tests or Fisher’s exact tests for categorical variables and Student’s t-tests for continuous variables

d: q-values were calculated using FDR (False Discovery Rate) correction

e: Significant variables after FDR correction showing q-value < 0.05

### Multivariate analysis

Proceeding to the multivariable logistic regression model (results shown in Table 3), adjusted for relevant clinical and exposure-related factors, we found that neither low platelet count (< 20 × 10³/µL) nor high viral load (Ct ≤ 25) remained independently associated with mortality after adjustment (adjusted OR 0.99; *p* = 0.059 and adjusted OR 0.93; *p* = 0.170, respectively). Of the clinical indicators, epistaxis was the only variable that retained a statistically significant association with mortality, with more than a four-fold increased adjusted odds (OR 4.70; 95% CI 1.08–20.4; *p* = 0.039). Other bleeding manifestations and gastrointestinal bleeding showed no independent effect, and jaundice produced an infinite confidence interval due to the small number of cases (five patients presented with jaundice at hospital admission, three of whom died; CFR = 60%), resulting in quasi-complete separation and limiting interpretability.

**Table 3:** Multivariable logistic regression analysis of predictors associated with increased mortality among confirmed CCHF Cases^a^

Laboratory Variable		OR^b^		95% CI^b^	*p*-value^c^
Low platelet count (< 20 × 10³/µL)		0.99		[0.98, 1]	0.059
High viral load (Ct ≤ 25)		0.93		[0.84–1.03]	0.170

**Clinical Variable**	**OR** ^**b**^		**95% CI** ^**b**^		**p-value** ^**c**^
**Epistaxis**	**4.70**		**[1.08**,** 20.4]**		**0.039**
Gastrointestinal bleeding	1.43		[0.41, 5.05]		0.580
Any bleeding signs	2.17		[0.74, 6.34]		0.157
Jaundice	101,230,522		0–Inf		0.99

a: Data source: Ministry of Health Iraq/Communicable Disease Control Center. *N* = 273 CCHF cases with known outcome, reported from 2021 to 2024

b: OR = Odds Ratio; CI = Confidence Interval

c: Comparisons were conducted using chi-square tests or Fisher’s exact tests for categorical variables and Student’s t-tests for continuous variables

**Fig. 1:**
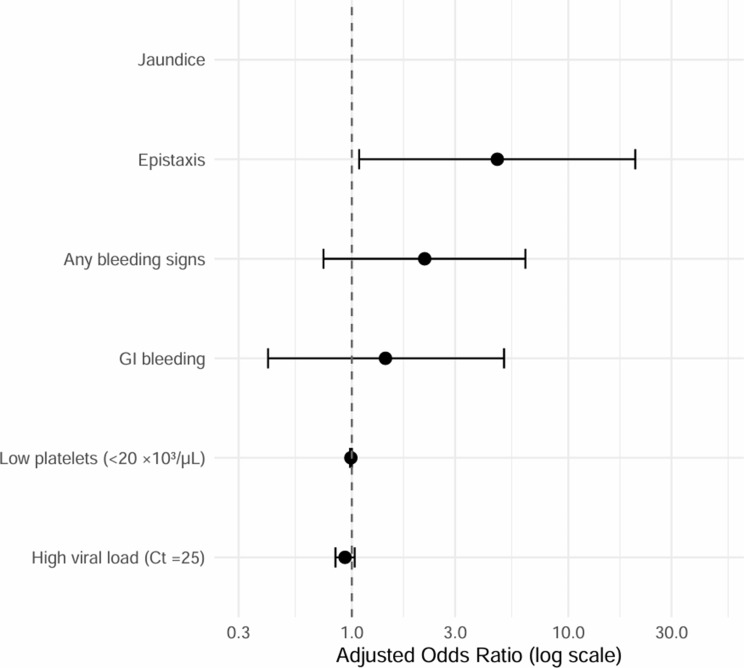
Forest plot for adjusted mortality predictors among confirmed CCHF cases^a.^ a: data source: ministry of health Iraq/communicable disease control center. *N* = 273 CCHF cases with known outcome, reported from 2021 to 2024. *GI bleeding=Gastrointestinal bleeding

The forest plot (Fig. 1) illustrates adjusted odds ratios (ORs) and 95% confidence intervals (CIs) for factors associated with mortality among confirmed CCHF patients.

### An alternate set of CCHF severity indicators for resource-limited settings

We first assessed how many indicators required by the CCHF-SSI (Crimean-Congo Hemorrhagic Fever Severity Scoring Index) [7] and SGS (Swanepoel’s Grading Score) [8] were available in our study cohort (total confirmed CCHF cases = 273). The SGS simplifies and aggregates many detailed indicators that are utilized in the SSI, whereas the SSI provides greater numerical precision, making it ideal for detailed research or systematic evaluations. The comparison between the indicators required by the CCHF-SSI and those available in our dataset from Iraq is presented in Table 4.

**Table 4:** CCHF SSI* vs. Availability of Iraq Data^a^

CCHF-SSI Indicator	CCHF-SSI Parameter	CCHF-SSI Score	Obtained Iraq Data (Total = 273)
Bleeding manifestations	None	0	269 (99%)
	Mild (petechiae, epistaxis)	1	
	Moderate/severe (GI, urinary, extensive skin)	2	
Altered mental status	Absent	0	273 (100%)
	Mild (confusion, lethargy)	1	
	Severe (coma, seizures)	2	
Hemodynamic instability	Stable	0	265 (97%)
	Hypotension (responding to fluids)	1	
	Shock (requiring vasopressors)	2	
Platelet count (×10³/µL)	> 100	0	256 (94%)
	20–100	1	
	< 20	2	
AST or ALT (U/L)	< 150	0	AST = 138 (51%) ALT = 139 (51%)
	150–500	1	
	> 500	2	
Creatinine (mg/dL)	< 1.5	0	26 (9.5%)
	1.5–3.0	1	
	> 3.0	2	
Prothrombin Time (PT)	Normal	0	127 (47%)
	Mildly prolonged	1	
	Severely prolonged (> 20 s)	2	

*Severity Classification (CCHF SSI total score): Mild: 0–3 points, Moderate: 4–7 points, Severe: ≥8 points. Severity is calculated by assigning points to each indicator

a: Data source: Ministry of Health Iraq/Communicable Disease Control Center. *N* = 273 CCHF cases with known outcome, reported from 2021 to 2024

As noted in the Methods, although information on altered mental status was recorded for all patients, its assessment at hospital admission relied on subjective clinical judgment without standardized or validated criteria across study sites. Given the multicentre nature of the dataset and the study’s aim to identify reproducible, objective bedside severity indicators suitable for resource-constrained settings, this variable was not included in the regression analyses. Considering data availability in Iraq and building upon the preliminary results presented in Tables 1, 2 and 3; Fig. 1, it was deemed suboptimal to include AST, ALT, creatinine, and prothrombin time (PT) in the CCHF severity scores adapted for resource-constrained settings, not because these parameters lack predictive accuracy for CCHF severity, but rather due to their limited availability.

**Fig. 2:**
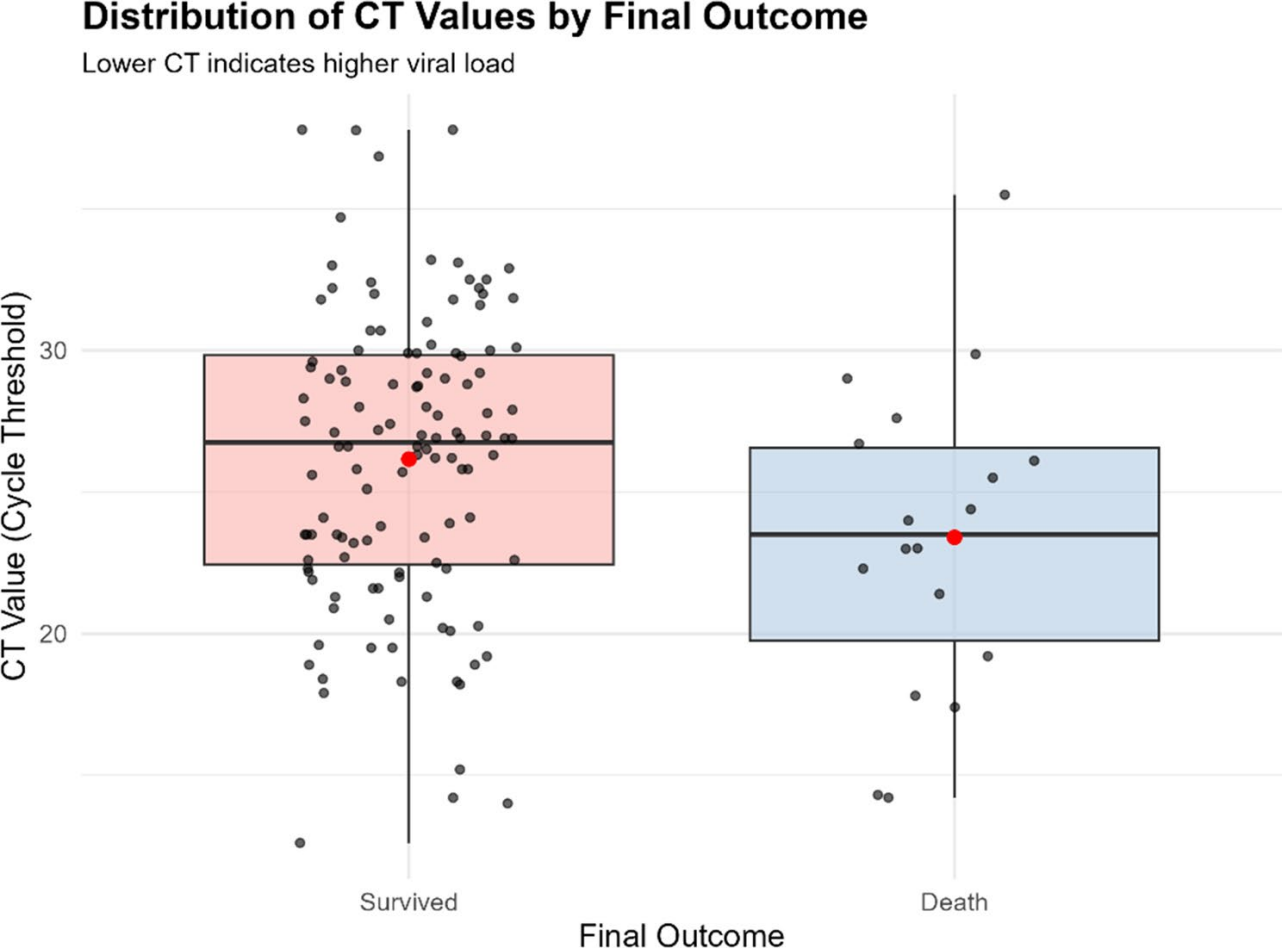
Distribution^*^ of Ct-values^a^ by final outcome. *Lower Ct-value indicates higher viral load, while higher Ct-value indicates lower viral load. a: Data source: Ministry of Health Iraq/Central Public Health Laboratory. *N* = 138 CCHF confirmed cases with Ct-value data, reported from 2021 to 2024

Based on the univariate and multivariable analyses, a simplified severity scoring system was developed using three variables: epistaxis, low platelet count (< 20 × 10⁹/L), and high viral load (Ct value ≤ 25). Although Ct value data are not routinely available and lost statistical significance in the multivariable analysis after adjustment, it was necessary to evaluate and compare its predictive accuracy, as lower Ct values indicate higher viral loads and were associated with increased mortality, whereas higher Ct values indicate lower viral loads and were associated with improved survival (Fig. 2).

**Fig. 3:**
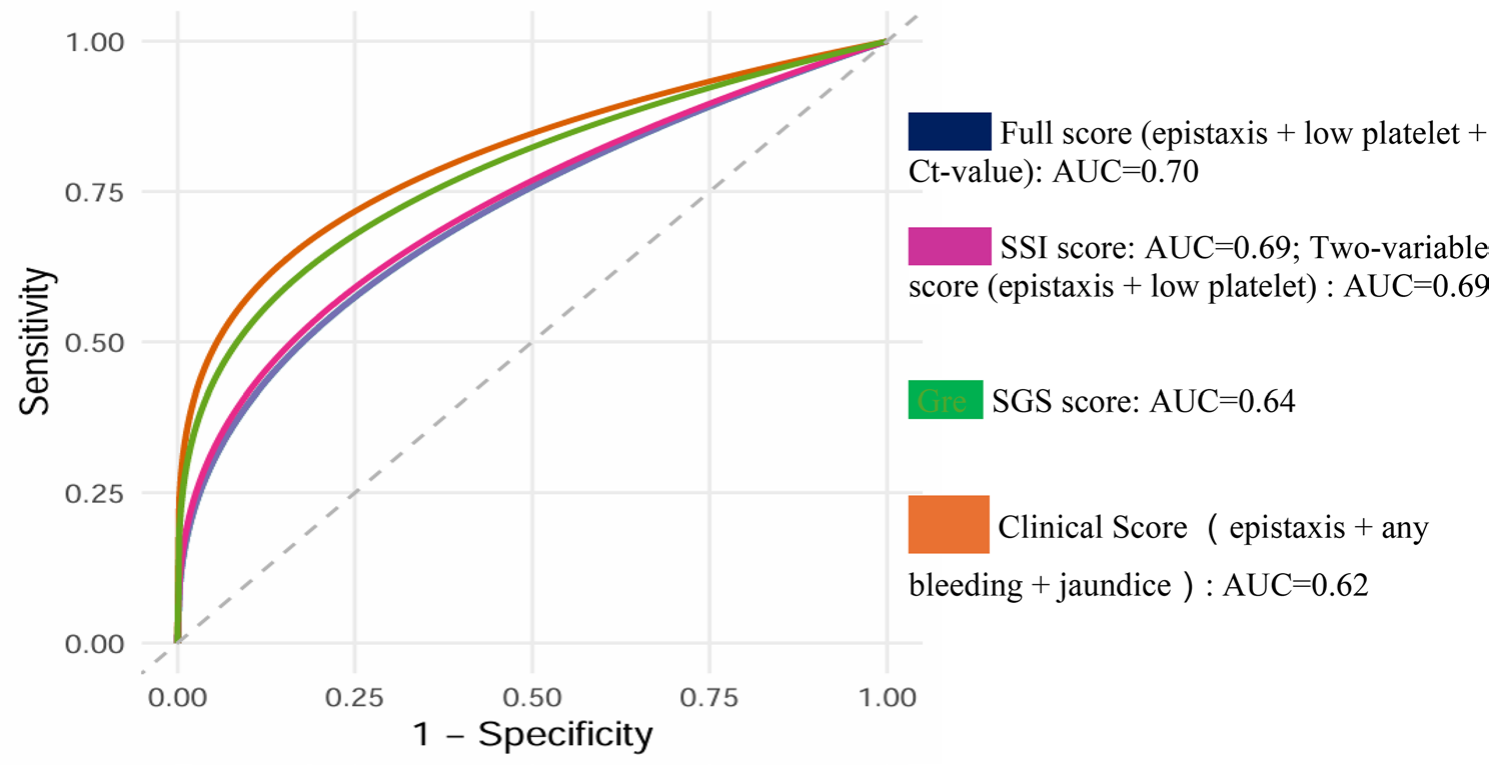
AUC* comparison of suggested scores vs. SSI* and SGS* scores. AUC*=The area under the curves comparing the predictive performance of the newly proposed CCHF severity score, the SSI, and the SGS in discriminating between survivors and non-survivors. SSI*= CCHF-SSI (Crimean-Congo Hemorrhagic Fever Severity Scoring Index). SGS*= Swanepoel’s Grading Score

Next, we removed the Ct value and developed a two-variable score using epistaxis and low platelet count. We then constructed a purely clinical three-variable score comprising epistaxis, any bleeding signs (including gastrointestinal bleeding), and jaundice, reflecting their practical availability across all levels of care and their relative importance in univariate and multivariable analyses. The predictive performance of each score was evaluated using Receiver Operating Characteristic (ROC) curve analysis and compared with existing SSI and SGS scores (Fig. 3). The Area Under the Curve (AUC) values, in order of highest accuracy, were: 0.70 for the suggested score combining epistaxis, low platelet count, and high viral load (Ct ≤ 25); 0.69 for the two-variable score (epistaxis and low platelet count); 0.69 for SSI; 0.64 for SGS; and 0.62 for the purely clinical three-variable score (epistaxis, any bleeding signs, and jaundice).

Taken together, these findings suggest that although laboratory-enhanced scores demonstrate the highest discriminatory performance, simpler models; such as the two-variable score (epistaxis + low platelet count) and the purely clinical score (epistaxis + any bleeding signs + jaundice); perform reasonably well and may offer meaningful practical advantages in low-resource settings where laboratory diagnostics are limited.

## Discussion

### Risk factors associated with mortality among confirmed CCHF cases

In this national cohort of 273 laboratory-confirmed CCHF cases in Iraq, the overall case fatality rate (CFR) was 12.1%, which falls within the mid-range of global estimates (5–40%) and underscores CCHF as a persistent public health threat. Variations in diagnostic sensitivity, reporting completeness, patient selection (hospital-based vs. national surveillance), and timely access to healthcare likely contribute to the observed CFR. Comparable or higher CFRs have been reported from other endemic settings, including Oman (~ 36.4%) [19], Iran (11–18%) [20], Afghanistan (36–42%) [21], and Pakistan (15–20%) [22]. These findings collectively highlight the ongoing regional and global need for improved triage, simplified severity assessment, and timely clinical intervention.

Most patients in this study resided in semi-urban or rural areas, and key occupational groups included homemakers, butchers, and animal owners, reflecting common exposure pathways through domestic animal care and slaughtering without appropriate protective measures. Similar patterns have been documented in Oman, where over 60% of CCHF cases occurred among individuals engaged in animal husbandry or butchering [19], and in Iran and Turkey, where farming and veterinary work are recognized as high-risk occupations [23, 24]. Although neither residence nor occupation was independently associated with mortality in our cohort, they remain critical for understanding transmission dynamics and targeting prevention. While female patients and those with delayed hospital admission showed descriptively higher case-fatality rates, these trends did not reach statistical significance, possibly reflecting limited power and heterogeneity in health-seeking behaviour and referral pathways. Nevertheless, the observation that admissions ≥ 10 days after symptom onset were associated with the highest CFR underscores the critical importance of early recognition and referral, particularly for women and in governorates with longer onset-to-admission intervals. It is also important to note that all parameters included in this analysis were collected at the time of hospital admission. Data on subsequent treatment and clinical care during hospitalization for the same 273 patients were also recorded in this study but were not included in the present analysis. These data—together with the potential impact of onset-to-admission delay on mortality, as presented in Supplementary Material 4—will be analyzed in subsequent research.

With respect to laboratory indicators, low platelet count and low Ct value (reflecting high viral load) were associated with mortality in descriptive and univariate analyses. The lowest platelet category (< 20 × 10³/µL) had the highest CFR (20.5%), and high viral load (Ct ≤ 25) was associated with nearly a three-fold increase in the odds of death. These findings are consistent with prior studies that identify thrombocytopenia and high viral load as markers of severe disease [13, 14]. However, after adjustment for clinical variables, neither platelet count nor Ct value remained independently associated with mortality (Table 3), suggesting that their prognostic effect may be largely mediated through clinically apparent hemorrhagic manifestations.

Among clinical variables, epistaxis emerged as the most robust mortality predictor. It remained significantly associated with death in multivariable analysis, conferring more than a four-fold increase in adjusted odds of mortality (OR 4.70; 95% CI 1.08–20.4). This reinforces previous evidence from Iraq and Turkey that mucosal bleeding, especially epistaxis, is a key marker of severe CCHF [6, 25, 26]. Gastrointestinal bleeding, petechiae, and the composite “any bleeding” variable showed strong associations in univariate models but lost significance after adjustment, likely due to overlap with epistaxis and the relatively small number of deaths. Focusing on a small set of specific hemorrhagic signs - particularly epistaxis - may therefore be more practical and informative for rapid severity assessment than monitoring a broad list of bleeding manifestations.

Jaundice was also strongly associated with mortality in univariate analysis (OR 11.9), and its presence likely reflects underlying hepatic dysfunction and multi-organ failure. Although less frequently emphasized in the CCHF literature, jaundice has been described as a late-stage complication with poor prognosis in some Middle Eastern cohorts [16, 27]. In our setting, routine measurement of liver enzymes (AST, ALT) was limited: AST and ALT were available in only about half of the cohort (138 and 139 patients, respectively), mainly due to restricted in-hospital laboratory capacity and cost constraints. In such contexts, jaundice may serve as a pragmatic clinical proxy for hepatic dysfunction and could be incorporated into simplified severity assessments where biochemical testing is unavailable.

Overall, our findings indicate that in this Iraqi cohort, mucosal hemorrhage (particularly epistaxis) and jaundice capture much of the prognostic information related to platelet count and viral load. Clinicians should treat these manifestations as red-flag indicators requiring urgent escalation of care, especially in facilities where laboratory diagnostics are delayed or limited.

### An alternate set of CCHF severity indicators for resource-constrained settings

Existing CCHF severity scores, such as the Crimean-Congo Hemorrhagic Fever Severity Scoring Index (CCHF-SSI) [7] and Swanepoel’s Grading Score (SGS) [8], use combinations of clinical and laboratory parameters to stratify disease severity and prognosis. However, our assessment of data availability in Iraq showed that key biochemical and coagulation markers required for these scores (e.g. AST/ALT, creatinine, prothrombin time) were missing for a large proportion of patients, especially in peripheral hospitals (Table 4). This reflects common challenges in endemic countries: inadequate laboratory infrastructure, supply shortages, and the cost and complexity of testing, all of which limit the routine application of comprehensive scores and risk under-recognition of severe cases.

Given these realities, we sought to identify a minimal yet clinically meaningful set of severity indicators that could be applied at the bedside in resource-constrained settings. Building on our univariate and multivariable analyses, we developed three simplified scores:

- Laboratory-enhanced three-variable score: epistaxis, low platelet count (< 20 × 10³/µL), and high viral load (Ct ≤ 25).
- Two-variable score: epistaxis and low platelet count, excluding Ct value to reflect settings without RT-PCR or Ct value reporting.
- Purely clinical three-variable score: epistaxis, any bleeding signs (including gastrointestinal bleeding), and jaundice.

When evaluated by ROC analysis against the SSI and SGS, the laboratory-enhanced three-variable score achieved an AUC of 0.70, comparable to the more complex SSI (AUC 0.69). The two-variable score (epistaxis + low platelet count) also performed well, with an AUC of 0.69, suggesting that the incremental benefit of including Ct value in this dataset was modest. The purely clinical score (epistaxis + any bleeding + jaundice) attained an AUC of 0.62, only slightly lower than SGS (0.64) (Fig. 3).

These results indicate that very simple combinations of a few key variables can approach the performance of more complex tools, while being far easier to implement in routine practice. Importantly, they can be applied at the point of first contact - even before laboratory results are available - enabling earlier risk stratification, prioritization of monitoring, and timely referral to higher-level care. In addition, this study strongly supports the routine reporting of Ct values from diagnostic laboratories to clinicians, as such data are generated automatically during RT-PCR and can be incorporated into severity assessment at negligible extra cost.

External validation in independent cohorts and prospective evaluation of their impact on clinical decision-making are essential next steps. In parallel, a simplified, risk-based triage algorithm that combines these clinical indicators with basic laboratory parameters, where available, could be developed for peripheral hospitals that lack comprehensive laboratory capacity.

Finally, although no specific antiviral therapy is currently licensed for CCHF, early identification of high-risk patients enables timely supportive care, including hemodynamic stabilization, blood products transfusion, organ support, and strict infection prevention and control measures, all of which are known to improve outcomes. Treatment and clinical-management data collected for the same 273 patients will be analysed separately to further refine case-management recommendations and support the design of future interventional studies.

### Limitations of the study

This study has several limitations. First, although Iraq reported 1,193 laboratory-confirmed CCHF cases between 2021 and 2024, complete standardized clinical and laboratory datasets were available for only 273 cases (23%). The majority of records were compiled retrospectively, and missing or incomplete forms limited inclusion. This partial coverage may have introduced selection bias, as patients treated at designated CCHF hospitals with better documentation could differ systematically from those managed elsewhere or during periods of system strain.

Second, substantial geospatial heterogeneity was observed in onset-to-admission times, ranging from 1 to 14 days across governorates (Supplementary Material 4). These differences likely reflect inequities in access to healthcare and variability in referral pathways, as well as differences in health-seeking behaviour. Delays in admission are likely to influence both symptom severity at presentation and mortality risk, yet could not be fully adjusted for in our models.

Third, missing data were common for several key laboratory variables, particularly Ct values (available for 51% of cases) and biochemical markers such as AST, ALT, creatinine, and prothrombin time, due to limited laboratory capacity and test availability. Incomplete laboratory data may have reduced statistical power and constrained our ability to fully evaluate established scores such as SSI and SGS.

Fourth, the simplified severity scores proposed were developed and internally validated using the same dataset, raising the possibility of overfitting and overly optimistic estimates of predictive performance. Although variable selection was guided by both statistical significance and biological plausibility, the relatively small number of deaths (*n* = 33) limited model complexity and resulted in wide—or, in the case of jaundice, infinite—confidence intervals due to quasi-complete separation. Because jaundice was observed in only a small number of patients, its association with mortality should be interpreted with caution. Larger multicentre studies are needed to refine model coefficients and assess reproducibility.

Fifth, residual confounding and misclassification cannot be excluded. Routine surveillance and hospital data are subject to variation in clinical assessment, under-reporting of symptoms, and differences in diagnostic practices.

Finally, as the analysis is based solely on patients from Iraq, generalizability to other CCHF-endemic regions may be limited. Differences in viral strains, tick ecology, host factors, healthcare infrastructure, and clinical management could influence disease presentation and outcomes. External validation in diverse settings is therefore essential.

## Conclusion

This Iraqi national study provides one of the most detailed assessments of Crimean-Congo hemorrhagic fever to date, integrating epidemiological, sociodemographic, laboratory, and clinical data from 273 laboratory-confirmed cases. The overall CFR of 12.1% confirms CCHF as a major public health concern, particularly in rural and high-exposure populations.

Our findings highlight epistaxis and jaundice as key clinical predictors of mortality, alongside low platelet count and high viral load on univariate analysis. In adjusted models, epistaxis remained the only independent predictor, emphasizing the prognostic importance of mucosal hemorrhage. Jaundice, though infrequent, likely reflects advanced hepatic dysfunction and may serve as a valuable clinical proxy where liver function tests are unavailable.

By systematically assessing the feasibility of existing severity scores and the availability of their required parameters, we demonstrated that simple, practical, and essential scores based on a small number of readily obtainable indicators - such as epistaxis, low platelet count, high viral load, and, where necessary, jaundice and other bleeding signs - can achieve discrimination comparable to more complex tools like SSI and SGS. The proposed two-variable (epistaxis + low platelet) and purely clinical scores (epistaxis + any bleeding + jaundice), while requiring external validation, offer practical options for bedside triage in resource-constrained settings where comprehensive laboratory testing is not feasible.

Implementation of a simplified CCHF triage checklist incorporating epistaxis and jaundice, and incorporating platelet count and Ct value where available, could help frontline healthcare providers rapidly identify and prioritize high-risk patients for closer monitoring and early, intensive supportive care. At the same time, strengthening public awareness, improving timely access to healthcare, standardizing the reporting of Ct values, and expanding laboratory and critical-care capacity will be essential components of a comprehensive CCHF control strategy. Continued surveillance, operational research, and collaboration between national authorities, international partners, and One Health stakeholders will be crucial for refining these tools, validating them in other endemic regions, and ultimately reducing CCHF-related mortality in Iraq and beyond.

## Supplementary Information

Below is the link to the electronic supplementary material.

Supplementary Material 1

## Data Availability

The datasets generated and analyzed during the current study are available from the corresponding author upon reasonable request. Data sources include the Iraqi Ministry of Health and the World Health Organization. Key data are also presented in the manuscript tables, figures, and supplementary materials.

## Abbreviations

ALT: Alanine Aminotransferase
AST: Aspartate Aminotransferase
CBC: Complete Blood Count
CCHF: Crimean-Congo Hemorrhagic Fever
CCHFV: Crimean-Congo Hemorrhagic Fever Virus
CFR: Case Fatality Rate
CI: Confidence Interval
CPHL: Central Public Health Laboratory
Ct-value: Cycle threshold value
DOH: Directorate of Health
ELISA: Enzyme-Linked Immunosorbent Assay
FDR: False Discovery Rate
GI bleeding: Gastrointestinal bleeding
IQR: Interquartile Range
OR: Odds Ratio
PT: Prothrombin Time
RT-PCR: Reverse Transcription Polymerase Chain Reaction
SGS: Swanepoel’s Grading Score
SSI (CCHF-SSI): Crimean-Congo Hemorrhagic Fever Severity Scoring Index
WHO: World Health Organization
