# Supplementary material for: Crimean-congo hemorrhagic fever in Iraq, 2021–2024: epidemiological and clinical data analysis with proposed severity indicators for resource-constrained settings
Source: BMC Infect Dis. 2026 Feb 2;26:481. doi: 10.1186/s12879-026-12759-z (PMC12955311; doi:10.1186/s12879-026-12759-z)
Supplement: Supplementary file 1 — Supplementary Material 1 [file 12879_2026_12759_MOESM1_ESM.docx]

**[Supplementary Materials]**

**Supplementary Material 1:**

**Comparison of laboratory confirmed CCHF cases^a^ from 2015-2020 to year 2025 in Iraq by month**


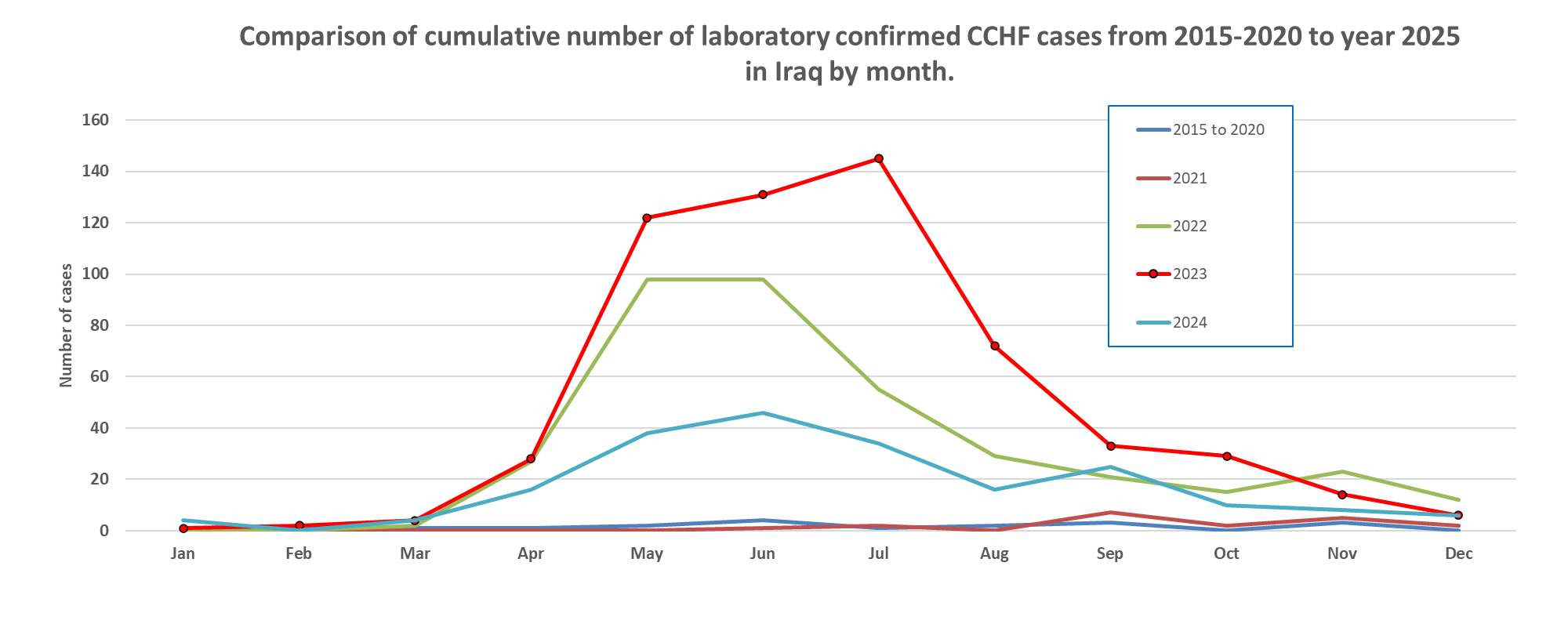


a: Data source: Ministry of Health Iraq/Communicable Disease Control Center. CCHF cases with known outcome, reported from 2015 to 2024.

**Supplementary Material 2:**

**Data collection form (original Arabic)**

**
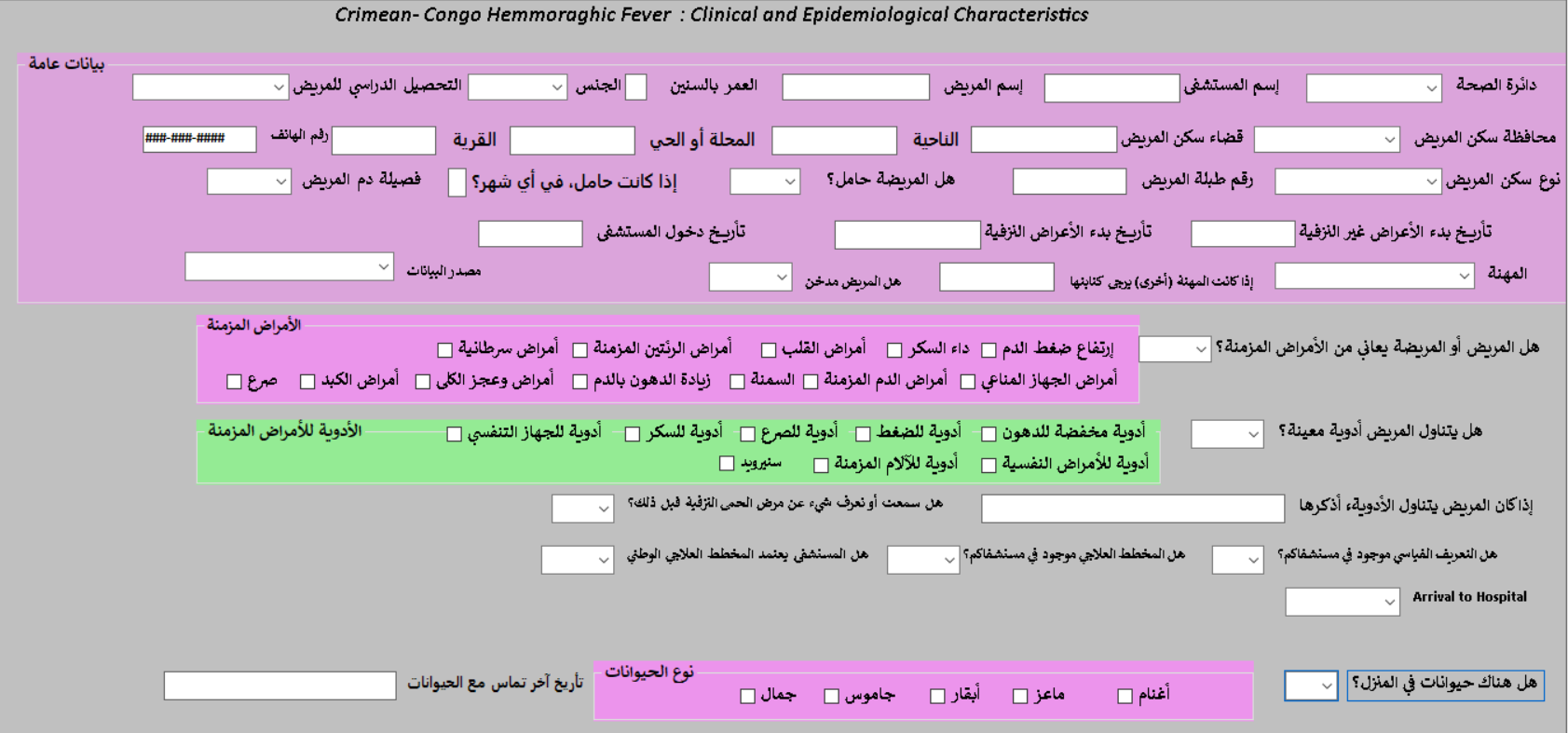
**

**The list of indicators collected (English)**

| **Indicators** |  |
| --- | --- |
| ID |  |
| Confirmed_case |  |
| ReportedDOH |  |
| HospitalName |  |
| PatientName |  |
| Age |  |
| Sex |  |
| Education |  |
| ResidencyProvince |  |
| District |  |
| Nahia |  |
| Mahalaa |  |
| Village |  |
| Kindofresidency |  |
| Case number |  |
| Pregnancy |  |
| IfPregnantinWhichMonth |  |
| BGroupRH |  |
| Occupation |  |
| Other (occupation) |  |
| D. Non bleeding onset |  |
| D. bleeding onset |  |
| onset_admission |  |
| DateofAdmission |  |
| Month |  |
| Year |  |
| Smoking |  |
| DataSource |  |
| Comorbidities |  |
| Hypertension |  |
| IschemicHeartDiseases |  |
| Epilepsy |  |
| Diabetes |  |
| Obesity |  |
| Malignant |  |
| ChronicobstructivePulmonaryDiseases |  |
| Other |  |
| Other Specify |  |
| Drughistory |  |
| Antihypertensive |  |
| CholesterolloweringDrug |  |
| AntiDiabetes |  |
| Anticonvulsions |  |
| Renal diseases |  |
| Steroid |  |
| Otherdrug history |  |
| Other drug history specify |  |
| Knowing CCHF |  |
| Presence of Case definition |  |
| Presence of treatment protocol |  |
| Using National ttt protocol |  |
| Patient arrival at hospital |  |
| Presence of Animal |  |
| Sheep |  |
| Goat |  |
| Cows |  |
| Buffalo |  |
| Camel |  |
| Other animal |  |
| Date of Contact with Animal |  |
| Presence of ticks on animals or at home |  |
| History of Tick Bite |  |
| Date of Tick Bite |  |
| Slaughtering history |  |
| Slaughtering date |  |
| Raw meat History |  |
| Raw meat Date |  |
| Family case contact history |  |
| CCHF case contact history |  |
| CCHF case contact date |  |
| Travel History within Last 14 days |  |
| Travel History Where if any |  |
| Fever |  |
| Headaches |  |
| Malaise |  |
| Joint Pain |  |
| Diarrhea |  |
| Vomiting |  |
| Abdominal Pain |  |
| Retro Orbital Pain |  |
| Eye Redness |  |
| Jaundice |  |
| Somnolence |  |
| Confusion |  |
| Others Specify |  |
| Ecchymosis |  |
| Petechiae |  |
| Bleeding from site of injection |  |
| Hepatomegally |  |
| Failure of organ |  |
| BleedingGums |  |
| Epistaxis |  |
| GIT Bleeding |  |
| Hematuria |  |
| Vaginal bleeding |  |
| Bleeding |  |
| Temp |  |
| PR |  |
| BP |  |
| Spo2 |  |
| Date of investigation |  |
| Inv_adm |  |
| Hemoglobin |  |
| WBC |  |
| Lymphocytes |  |
| Neutrophils |  |
| Platelets |  |
| LDH |  |
| TSB |  |
| Fibrinogenlevel |  |
| CRP |  |
| esr |  |
| DDimer |  |
| SAlbumin |  |
| PT |  |
| INR |  |
| PTT |  |
| aPTT |  |
| ALT |  |
| AST |  |
| Ferritin |  |
| RBS |  |
| HBA1C |  |
| Burea |  |
| Fibrinogen Score |  |
| Platletes Score |  |
| aPTT score |  |
| Bleeding Score |  |
| Somnolence Score |  |
| SSI |  |
| Final Scoring SSI |  |
| Full_SSI |  |
| ASTs |  |
| ALTs |  |
| LDHs |  |
| WBCs |  |
| Hepatomegaly s |  |
| organ failuare s |  |
| Bleeding-S |  |
| Age-S |  |
| Platelets-S |  |
| PT-S |  |
| aPTT-S |  |
| INR-S |  |
| SGS |  |
| Final Score SGS |  |
| Full_SGS |  |
| Date of Sample taken |  |
| PCR |  |
| CT |  |
| CT_group |  |
| Lab2f |  |
| Follow up test date |  |
| HemoglobinF |  |
| PlateletsF |  |
| WBCF |  |
| PTF |  |
| PTTF |  |
| FibrinogenlevelF |  |
| CRPF |  |
| RBSF |  |
| BureaF |  |
| TSBF |  |
| ALTF |  |
| ASTF |  |
| Lab3 |  |
| Lab3 date |  |
| Hgb3 |  |
| Platelets3 |  |
| WBC3 |  |
| RBC3 |  |
| ALT3 |  |
| AST3 |  |
| TSB3 |  |
| PT3 |  |
| PTT3 |  |
| Sodium |  |
| Potassium |  |
| Creatinine |  |
| Chloride |  |
| INR3 |  |
| Burea3 |  |
| RBS.1 |  |
| Glucose |  |
| ESR |  |
| Others investigations Done1 |  |
| Other investigation type1 |  |
| Others investigations results1 |  |
| Others investigations date1 |  |
| Other investigation type2 |  |
| Others investigations results2 |  |
| Others investigations date2 |  |
| Other investigation type3 |  |
| Others investigations results3 |  |
| Others investigations date3 |  |
| Supportive Therapy |  |
| IV Fluids |  |
| IV fluids dose in CC |  |
| IV fluids start date |  |
| IV fluids cessation date |  |
| Antipyeritic |  |
| Antipyeritic dose in mg |  |
| Antipyeritic start date |  |
| Antipyeritic cessation date |  |
| PPIs |  |
| PPIs dose in mg |  |
| PPIs start date |  |
| PPIs cessation date |  |
| Anti-emetic |  |
| Anti-emetic dose in mg |  |
| Anti-emetic start date |  |
| Anti-emetic cessation date |  |
| Cyklokapron |  |
| Cyklokapron dose in mg |  |
| Cyklokapron start date |  |
| Cyklokapron cessation date |  |
| Others Type |  |
| Other supportive dose |  |
| Other Supportive date |  |
| Other Cessation date |  |
| Blood and Bloodproduct |  |
| Platelets transfusion |  |
| Platelets  amount in pints |  |
| Platelets date |  |
| Platelets cessation |  |
| Plasma transfusion |  |
| Plasma amount in pints |  |
| Plasma date |  |
| Plasma cessation |  |
| RBCs transfusion |  |
| RBCs amount in pints |  |
| RBCs date |  |
| RBCs cessation |  |
| Cryoprecipitate |  |
| Cryoprecipitateamount |  |
| Cryoprecipitatedate |  |
| Cryoprecipitatecessationdate |  |
| Antibiotics usage |  |
| Antibiotic1type |  |
| Antibiotic1dose in mg |  |
| Antibiotic1formula |  |
| Antibiotics1date |  |
| Antibiotic1cessation |  |
| Antibiotic2type |  |
| Antibiotic2dose |  |
| Antibiotic2formula |  |
| Antibiotics2date |  |
| Antibiotic2cessation |  |
| Name of Antibiotic3 |  |
| DosesofAntibiotic3 |  |
| Antibiotic3formula |  |
| Date of Antibiotics3 used |  |
| Date of Cessation antibiotic3 |  |
| rib_onset |  |
| ribavarin_usage |  |
| Ribavarin1dose in mg |  |
| Ribavirin1type |  |
| Ribavarin1date |  |
| Ribavirin1cessation |  |
| Ribavarin2dose in mg |  |
| Ribavarin2type |  |
| Ribavarin2date |  |
| Ribavarin2cessation |  |
| ribavarin_duration |  |
| rib_admission |  |
| Total dose |  |
| av_daily_rib_dose |  |
| main_rib_type |  |
| Steriods case ttt |  |
| Steroid1type |  |
| Steroid1dose in mg |  |
| Steroid1formula |  |
| Steroid1date |  |
| Steroid1cessation |  |
| Steroid2type |  |
| Steroid2dose in mg |  |
| Steroid2date |  |
| Steroid2 cessation |  |
| Complications |  |
| Peripheral Neuropathy |  |
| Bradycardia |  |
| Behavior change |  |
| Depression |  |
| Hyperglycemia |  |
| Hallucination |  |
| Carditis |  |
| Renal Failure |  |
| Liver Failure |  |
| BleedingofBodyorfices |  |
| IntraAlveolarHemorrage |  |
| Secondary Infection |  |
| Pleural Effusion |  |
| Agitation |  |
| Seizures |  |
| ARDS |  |
| Liver failure |  |
| FinalDiagnosis |  |
| IF Diagnosis is not CCHF Mention it |  |
| Final_outcome |  |
| Date of outcome |  |
| If outcome is death Mention cause of death |  |
| IncubationPeriodinDays |  |
| IPA |  |
| IPS |  |
| IPM |  |
| IPT |  |
| IPC |  |
| DaysIgM |  |
| DaysIgG |  |
| PCR_adm |  |
| DaysPCR |  |
| Difference in Days between no bleeding symptoms and date of bleeding |  |
| Days between Date of onset OF no bleeding symptoms and date of investigation |  |
| Difference Between Date of investigation and Date of Bleeding signs |  |
| Days between Investigation and followup Tests |  |
| Stay in Hospital |  |
| Name of Data entry |  |
| PhyscianName |  |

**Supplementary Material 3:**

**Age Group and Baseline Profile among 273 Laboratory-Confirmed CCHF Cases in Iraq^a^**

| **Age Group**  **(Years)** | **Survived**  **N=240** | **Survived Median Age (IQR^b^)** | **Death**  **N=33** | **Death Median Age (IQR^b^)** | **p-value^c,d^** |
| --- | --- | --- | --- | --- | --- |
| <20 | 27 | 17.0 (14-20) | 3 | 18 (17-18) | 0.207256 |
| 20-39 | 118 | 30.0 (25-35) | 15 | 30 (25-34.5) | 0.726513 |
| 40-59 | 66 | 47.5 (42.5-52.5) | 12 | 47 (45-49) | 0.823882 |
| ≥60 | 28 | 64.5 (59.5-69.5) | 3 | 64 (62-66) | 0.80921 |
| N/A^*^ | 1 | N/A* | 0 | N/A* | N/A* |

| **Baseline Profile** | **All**  ***N=273*** | **Survived**  ***N=240*** | **Death**  ***N=33*** | **p-value^c,d^ overall** |
| --- | --- | --- | --- | --- |
| **Gender** | N*=273 (100%) | 240 | 33 | 0.146 |
| Female | 105 (38.5%) | 88 (36.7%) | 17 (51.5%) |  |
| Male | 168 (61.5%) | 152 (63.3%) | 16 (48.5%) |  |
| **Type of Residency** | N*=273 (100%) | 240 | 33 | 0.246 |
| Rural | 86 (31.5%) | 77 (32.1%) | 9 (27.3%) |  |
| Semi-urban | 113 (41.4%) | 95 (39.6%) | 18 (54.5%) |  |
| Urban | 73 (26.7%) | 67 (27.9%) | 6 (18.2%) |  |
| N/A* | 1 (0.4%) | 1 (0.4%) | 0 (0.0%) |  |
| **Animal Owner** | N*=273 (100%) | 240 | 33 | 1.000 |
| No | 246 (90.1%) | 216 (90.0%) | 30 (90.9%) |  |
| Yes | 27 (9.9%) | 24 (10.0%) | 3 (9.1%) |  |
| **Butcher** | N*=273 (100%) | 240 | 33 | 0.783 |
| No | 237 (86.8%) | 209 (87.1%) | 28 (84.8%) |  |
| Yes | 36 (13.2%) | 31 (12.9%) | 5 (15.2%) |  |
| **Homemaker** | N*=273 (100%) | 240 | 33 | 0.178 |
| No | 189 (69.2%) | 170 (70.8%) | 19 (57.6%) |  |
| Yes | 84 (30.8%) | 70 (29.2%) | 14 (42.4%) |  |
| **Presence of Animal** | N*=273 (100%) | 240 | 33 | 0.760 |
| No | 118 (43.4%) | 105 (43.9%) | 13 (39.4%) |  |
| Yes | 154 (56.6%) | 134 (56.1%) | 20 (60.6%) |  |
| N/A* | 1 (0.4%) | 1 (0.4%) | 0 (0.0%) |  |
| **History of Tick Bite** | N*=255 (93%) | 240 | 33 | 0.230 |
| No | 241 (94.5%) | 210 (93.8%) | 31 (93.9%) |  |
| Yes | 14 (5.5%) | 14 (5.8%) | 0 (0.0%) |  |
| N/A* | 18 (6.6%) | 16 (6.7%) | 2 (6.06%) |  |
| **Presence of ticks on animals or at home** | N*=273 (100%) | 240 | 33 | 0.700 |
| No | 212 (77.7%) | 186 (77.5%) | 26 (78.8%) |  |
| Yes | 45 (16.5%) | 41 (17.1%) | 4 (12.1%) |  |
| N/A* | 16 (5.9%) | 13 (5.4%) | 3 (9.1%) |  |
| **Slaughtering history** | N*=273 (100%) | 240 | 33 | 0.316 |
| No | 181 (66.3%) | 162 (68.1%) | 19 (57.6%) |  |
| Yes | 90 (33.0%) | 76 (31.9%) | 14 (42.4%) |  |
| N/A* | 2 (0.7%) | 2 (0.8%) | 0 (0.0%) |  |
| **Exposure to raw meat** | N*=273 (100%) | 240 | 33 | 0.580 |
| No | 164 (60.1%) | 142 (59.2%) | 22 (66.7%) |  |
| Yes | 106 (38.8%) | 95 (39.5%) | 11 (33.3%) |  |
| N/A* | 3 (1.1%) | 3 (1.3%) | 0 (0.0%) |  |
| **Comorbidities** | N*=273 (100%) | 240 | 33 | 0.094 |
| No | 239 (87.5%) | 207 (86.3%) | 32 (97.0%) |  |
| Yes | 34 (12.5%) | 33 (13.8%) | 1 (3.0%) |  |

a: Data source: Ministry of Health Iraq/Communicable Disease Control Center. *N*=273 CCHF cases with known outcome, reported from 2021 to 2024.

b: IQR=Interquartile Range

c: Comparisons were conducted using chi-square tests or Fisher’s exact tests for categorical variables and Student's t-tests for continuous variables.

d: A two-sided p-value of <0.05 was considered statistically significant.

N*=Actual number of collected data out of total 273 confirmed CCHF cases

N/A*=Data not available

**Supplementary Material 4:**

**4-1: Median Time from Symptom Onset to Hospital Admission by Governorate**

**4-2: Geospatial Differences in Onset to Admission Time by Governorates^a^**

| **Reported DOH^b^** | **Confirmed CCHF Cases with Available Data*** | **Onset to Admission (days),**  **Median (IQR^c^)** |
| --- | --- | --- |
| Babylon | 30 | 6 (5-7) |
| Baghdad | 16 | 5 (4-6) |
| Basrah | 11 | 5 (4-6) |
| Dahuk | 5 | 5 (3-6) |
| Diyala | 1 | 14 (14-14) |
| Erbil | 7 | 4 (2-5) |
| Kirkuk | 17 | 4 (2-5) |
| Missan | 5 | 5 (4-6) |
| Ninewa | 25 | 3 (2-4) |
| Sulamaniyah | 5 | 6 (3-6) |
| Thiqar | 118 | 3 (2-5) |
| Wassit | 38 | 3 (1-4) |

a: Data source: Ministry of Health Iraq/Communicable Disease Control Center. N=273 CCHF cases with known outcome, reported from 2021 to 2024.

b: DOH: Directorate of Health which represents the main local health authority responsible for overseeing and managing health services at the provincial (governorate) level in Iraq.

c: IQR=Interquartile Range

* Only data with available information was included in this analysis, and this is not representing the total number of confirmed CCHF cases from each governorate over the same time period.

**4-3: Time to Hospital Admission vs Mortality among 273 Laboratory-Confirmed CCHF Cases in Iraq^a^**

| **Time From Symptom Onset to Hospital Admission** | **All**  ***N=273* (Male/Female)** | **Survived**  ***N=240***  **(Male/Female)** | **Death**  ***N=33***  **(Male/Female)** | **CFR^e^ (%)**  **(Male CFR/**  **Female CFR)** | **p-value^c,d^** |
| --- | --- | --- | --- | --- | --- |
| 0–2 days | 68 (43/25) | 61 (40/21) | 7 (3/4) | 10.3 (7/16) | 0.751 |
| 3–5 days | 119 (72/47) | 104 (65/39) | 15 (7/8) | 12.6 (9.7/17) | 0.967 |
| 6–9 days | 52 (37/15) | 46 (33/13) | 6 (4/2) | 11.5 (10.8/13.3) | 1.000 |
| ≥10 days | 9 (6/3) | 7(5/2) | 2 (1/1) | 22.2 (16.7/33.3) | 0.668 |
| N/A* | 25 (10/15) | 22 (9/13) | 3 (1/2) | 12.0 (10/13.3) | N/A* |

a: Data source: Ministry of Health Iraq/Communicable Disease Control Center. *N*=273 CCHF cases with known outcome, reported from 2021 to 2024.

b: CFR=Case Fatality Rate

c: Comparisons were conducted using chi-square tests or Fisher’s exact tests for categorical variables and Student's t-tests for continuous variables.

d: A two-sided p-value of <0.05 was considered statistically significant.
